# Electrochemical Assays for the Determination of Antidiabetic Drugs—A Review

**DOI:** 10.3390/mi15010010

**Published:** 2023-12-20

**Authors:** Katarzyna Fendrych, Anna Górska-Ratusznik, Joanna Smajdor

**Affiliations:** 1 Faculty of Materials Science and Ceramics, AGH University of Krakow, al. Mickiewicza 30, 30-059 Cracow, Poland; 2 Lukasiewicz Research Network—Krakow Institute of Technology, 73 Zakopianska St., 30-418 Krakow, Poland

**Keywords:** diabetes, voltammetry, electrochemistry, working electrodes

## Abstract

This article presents the current state of knowledge regarding electrochemical methods for determining the active substances within drugs that are used in the treatment of type 1 and type 2 diabetes. Electrochemical methods of analysis, due to their sensitivity and easiness, are a great alternative to other, usually more expensive analytical assays. The determination of active substances mentioned in this review is based on oxidation or reduction processes on the surface of the working electrode. A wide variety of working electrodes, often modified with materials such as nanoparticles or conducting polymers, have been used for the highly sensitive analysis of antidiabetic drugs. The presented assays allow us to determine the compounds of interest in various samples, such as pharmaceutical products or different human bodily fluids.

## 1. Introduction

Diabetes mellitus is defined as a group of metabolic disorders manifesting with hyperglycemia (elevated glucose levels in the blood) caused by defects in insulin secretion or action. The literature describes three main types of diabetes: type 1 diabetes mellitus (T1DM), type 2 diabetes mellitus (T2DM) and gestational diabetes. Statistics show that the most common type of diabetes is T2DM, also called non-insulin-dependent diabetes. According to the WHO (World Health Organization), approximately 422 million people worldwide suffer from diabetes, with over 95% being cases of T2DM [1,2,3].

T1DM is considered an autoimmune disease, typically diagnosed in children and young adults. The disease is initiated when a patient’s immune system starts to destroy the β cells that are responsible for the regulation of glucose levels in the blood. The treatment of T1DM involves regular subcutaneous injections of insulin via a syringe or insulin pump, which should mimic normal pancreatic function. Non-insulin drugs are also used in T1DM therapy: amylin, pramlintide, metformin, GLP-1 receptor agonists and dipeptidyl peptidase-4 inhibitors [4,5,6].

In T2DM, a patient’s body does not produce enough insulin and/or cells do not respond properly to the insulin, which leads to increased blood glucose levels. Unlike T1DM, it affects mostly adults. According to the literature, the development of the disease is caused by both genetic and environmental factors. Therapy for T2DM involves, above all, a change in lifestyle by means of introducing a healthy diet and physical activity. Nevertheless, in most patients, pharmacological treatment is also necessary. The following groups of medications are recommended: sulfonylurea derivatives, dipeptidyl peptidase-4 inhibitors, biguanide derivatives, GLP-1 receptor agonists, sodium–glucose cotransporter-2 inhibitors and α-glucosidase inhibitors. In some patients, therapy based on insulin may be used as well [7,8,9].

The third common type of diabetes is gestational diabetes. It can develop during pregnancy in women who do not already suffer from diabetes. Pregnant women’s bodily changes include changing cell responses to the hormone that leads to the development of insulin resistance. According to recommendations, the treatment of gestational diabetes should be based on insulin injections [10,11,12,13].

Diabetes therapy most frequently involves the need to take medications on a daily basis. The effective control of glucose levels in the blood is crucial for the proper functioning and safety of patients. Therefore, the problem of quality control of the medications available on the pharmaceutical market is an important issue [14].

After administration, drugs are metabolized in a patient’s body, excreted and then end up in sewage. In this context, a higher consumption of medications raises a new problem. Relatively high quantities of organic substances of medical origin end up in sewage. Unfortunately, the methods utilized in sewage treatment plants are not sufficient enough to remove that kind of contamination. After treatment, sewage is released to rivers, which means that, eventually, different types of organic compounds end up in the environment. Therefore, the branch of analytical chemistry is still growing in the search for new methods of disposing of drugs and determining their metabolites.

Electrochemical methods are a useful tool in qualitative and quantitative drug determination, especially those discussed in this work, which include voltammetry and amperometry. In these methods, the analytical signal is related to the redox reaction of the analyte on the surface of the working electrode. The electrons generated during the reaction initiate current flow, the quantity of which is proportional to the amount of the analyte in the system.

The most important part of each voltametric and amperometric measurement system is the working electrode. Different constructions and materials are used depending on the type of analyte, measurement conditions, etc. The most traditional and the oldest types of sensors are mercury electrodes. Taking into account the toxicity of the metal, modern constructions aim to limit the consumption of mercury while maintaining the benefits of using the mentioned metal as an electrode material. One example of such construction might be a renewable amalgam film electrode. The other group of working electrodes are solid electrodes, including metal electrodes (e.g., Ag, Au, Pt), glassy carbon electrodes (GCEs), carbon paste electrodes (CPEs), screen-printed electrodes (SPEs), graphite-based electrodes or boron-doped diamond electrodes (BDDEs). The current trend in electrochemistry is focused on modifications of solid electrodes (mostly GCEs, CPEs, SPEs) with surface modifiers. Such materials should be characterized by good electrical conductivity, a high surface area or electrocatalytic properties. The aim of surface modification is to improve the sensitivity and selectivity of the electrode.

Electrochemical methods are utilised for determination of both organic and inorganic substances in samples characterised by simple and complex matrices. It is a versatile tool characterised by relatively high precision and accuracy. The cost of purchasing and maintaining equipment is relatively low. Another advantage of voltammetry is the possibility to measure very low concentrations of an analyte. The limits of detection (LODs) for voltametric methods are often comparable to those achieved using more complex and more expensive chromatographic methods. Voltametric and amperometric measurements do not require toxic solvents and the consumption of chemicals is very low, which is consistent with principles of green chemistry.

Considering the above-mentioned problem of the increasing consumption of antidiabetic drugs and environmental pollution, it is extremely important to develop analytical methods that will allow for the monitoring of the quality of produced pharmaceuticals, as well as their presence in the environment. Electrochemical methods are a versatile and useful tool in drug analysis; therefore, this review is devoted to voltametric and amperometric assays of antidiabetic drugs.

## 2. Electrochemical Measurements of Antidiabetic Drugs

### 2.1. Insulin

Insulin is an anabolic peptide hormone with systemic action, which plays an essential role in the metabolism of carbohydrates, proteins and fats. Insulin is secreted by the endocrine part of the pancreas, more specifically by the beta cells of the Langerhans islets, which constitute 75% of all islet cells. This hormone belongs to the group of peptide hormones and is created as a result of the combination of 51 amino acid residues. The insulin molecule consists of two polypeptide chains, alpha (α) and beta (β). Insulin participates directly or indirectly in all links in the metabolism of carbohydrates, proteins and fats. The most important stimulus for insulin secretion is an increase in blood glucose concentration. Most body cells have insulin receptors on their surface. In type I diabetes, insulin treatment is necessary because the pancreas does not produce this hormone at all. However, with type II diabetes, patients may be able to maintain normal blood glucose levels with appropriate diet, exercise and antidiabetic medications. Only when these methods do not deliver the desired results is insulin introduced into the treatment [15,16,17,18,19,20].

Interest in the possibility of insulin electrochemical quantification is very high; therefore, a lot of assays have been presented in the literature. Most of them use some kind of modified electrodes as the sensing platform (Table 1). As the most popular modifier, carbon nanotubes are used, along with the metal nanoparticles or metal oxides [21,22,23,24]. One of the lowest insulin detection limits was obtained using screen-printed platinum electrodes modified with molecularly imprinted polymer nanoparticles (NanoMIP/SPPE, LOD 26 fM) [25], and on the silver nanoflower-decorated, reduced graphene oxide-modified micro-disk electrode arrays (AgNF/rGO/MDEA, LOD 70 pg mL^−1^) [26]. Insulin has also been successfully measured both in pharmaceutical formulation and in real samples with complex matrices, such as human or animal bodily fluids (e.g., serum, plasma, urine).

### 2.2. Sulfonylurea Class of Antidiabetic Agents

**Figure 1:**
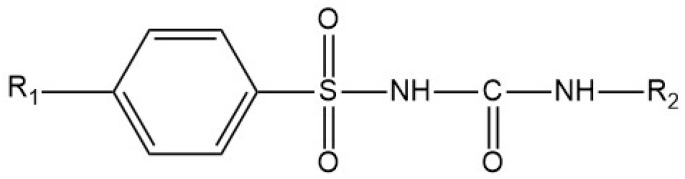
General structure formula of sulfonylureas.

Sulfonylureas (SUs) constitute the oldest class of drugs used for the treatment of type 2 diabetes mellitus, commercialized in the late 1950s. All pharmacological SUs contain a phenyl-sulphonyl-urea structure (Figure 1) with a p-substituent on the phenyl ring (R1) and various groups terminating the urea N end group (R2), which modulates their pharmacokinetic and pharmacotoxicological profile [14].

Sulfonylureas are widely used to maintain an appropriate blood glucose level in patients with type II non-insulin-dependent diabetes mellitus. As a group of insulin secretagogues, SUs stimulate endogenous insulin release from beta cells of the pancreas regardless of blood glucose levels [52]. SUs also lead to increased glucose uptake and oxidation and decreased liver gluconeogenesis, and may cause increased insulin receptor numbers and sensitivity. The most common effect connected with the mechanism of action of SUs is hypoglycemia [53].

Currently, several sulfonylureas are available for the treatment of type II diabetes mellitus. They are traditionally divided into two groups or generations of agents (Table 2). The first-generation sulfonylureas include chlorpropamide and tolbutamide, whereas gliclazide, glipizide, glibenclamide and glimepiride are second-generation sulfonylureas. As a result of being more potent and allowing administration at a lower, once-daily dose, the second-generation sulfonylureas have largely replaced the first-generation agents.

#### 2.2.1. Gliclazide

For the voltametric determination of gliclazide (GLZ), different kinds of working electrodes have been implemented (Table 3). A CPE-based sensor, which exhibited a linear response of GLZ in the range of 5 × 10^−7^–1.25 × 10^−6^ mol L^−1^, with the LOD value equal to 1 × 10^−7^ mol L^−1^, was successfully applied in the determination of GLZ in tablets [54]. An improvement in the analytical performance of GLZ sensors was achieved through the application of various sensing materials, including an electropolymerized molecularly imprinted polymer (E-MIP) [55], magnetic core–shell Fe_3_O_4_@SiO_2_ and multiwalled carbon nanotubes (MWCNTs) [56], ZnIn_2_S_4_ nanoparticles [57], MoWS2 [58] and magnetic core–shell manganese ferrite nanoparticles (MCSNPs) [59] in the modification of GCEs, CPEs and SPEs.

The high synergetic activity of ZnIn_2_S_4_ and ionic liquid (1-butyl-3-methylimidazolium hexafluorophosphate (BMIM.PF6)) resulted in the fabrication of a modified electrode (ZISILCPE) characterized by a wide linear range (7.5 × 10^−7^–5.0 × 10^−4^ mol L^−1^), low LOD (1.2 × 10^−7^ mol L^−1^) and applicability for the detection of GZL in pharmaceutical and urine samples in the presence of glibenclamide [57]. A simple, portable and sensitive sensor based on a MoWS2-modified screen-printed electrode (MoWS2/SPE) was fabricated and applied to the determination of GZL in biological and pharmaceutical samples. The utilization of a MoWS2 nanoparticle in the surface modification of the SPE resulted in an improvement in electron transfer rates, and thus an increase in sensitivity, which allowed one to obtain the LOD value of 1.8 × 10^−8^ mol L^−1^ [58].

The most favorable analytical performances in terms of the precision, selectivity and sensitivity of GLZ voltametric determination was exhibited by the sensor-based GCE modified with an electropolymerized molecularly imprinted polymer (E-MIP) film. The obtained excellent LOD value of 1.2 × 10^−11^ mol L^−1^ resulted from the easier and faster accessibility of recognition sites due to the very thin structure of the sensing layer [55]. 

#### 2.2.2. Glipizide

Only a few reports regarding the voltametric determination of glipizide (GLP) have been described so far (Table 4) [61,62,63,64]. As the working electrodes, CPEs, HMDEs and in situ-plated lead firm electrodes were used. By applying a simple and precision square-wave adsorptive stripping voltametric technique, it was possible to develop a procedure of GLP determination with an advantageous limit of detection equal to 1.5 × 10^−10^ mol L^−1^ and 2.5 × 10^−10^ mol L^−1^ achieved for the HMDE and the lead film electrode, respectively. The presented sensors were successfully applied for the quantitation of glipizide in pharmaceutical formulations and human urine samples.

#### 2.2.3. Glibenclamide

As shown (Table 5), the electroanalytical determination of glibenclamide (GBC) through the voltametric methods can be performed with the use of HMDEs [65], Sephadex-modified carbon paste electrodes (SMCPEs) [66], and ZnIn_2_S_4_ nanoparticles with ionic liquid-modified carbon paste electrodes (ZISILCPEs) [57]. The strong binding between GBC molecules and the Sephadex polymer at the surface of SMCPEs allowed for the analysis of trace levels of glibenclamide by means of a stripping analysis. As a result, the GBC sensor with linear range of 1.0 × 10^−9^–5.0 × 10^−8^ mol L^−1^ and an LOD of 4 × 10^−10^ mol L^−1^ was developed and applied to for determination of glibenclamide in commercially available antidiabetic drugs and human serum [66]. A square-wave adsorptive cathodic stripping (SW-AdCS) voltametric procedure for the quantification of GBC with a similar analytical performance (linear range of 2 × 10^−8^–1 × 10^−6^ mol L^−1^ and LOD of 6 × 10^−9^) was developed with the Hg electrode [65].

#### 2.2.4. Glimepiride

As reported in the literature, several papers have been published for the electrochemical detection of glimepiride (GLI) using various kinds of working electrode (Table 6). The vast majority of GLI voltametric sensors are based on the utilization of carbon-based electrodes, including carbon paste electrodes and glassy carbon electrodes [67,68,69], as well as hanging drop mercury electrodes [70,71,72,73]. These papers have focused on understanding the electrochemical behavior of GLI at a particular sensor and its quantitative determination, either alone or in combination with other antidiabetic drugs. By applying various types of voltametric techniques (DPV, SWV, SW AdCSV), the limit of GLI detection in the range of 2 × 10^−7^ mol L^−1^ to 1.7 × 10^−5^ mol L^−1^ was achieved under optimized conditions.

### 2.3. Metformin

**Figure 2:**
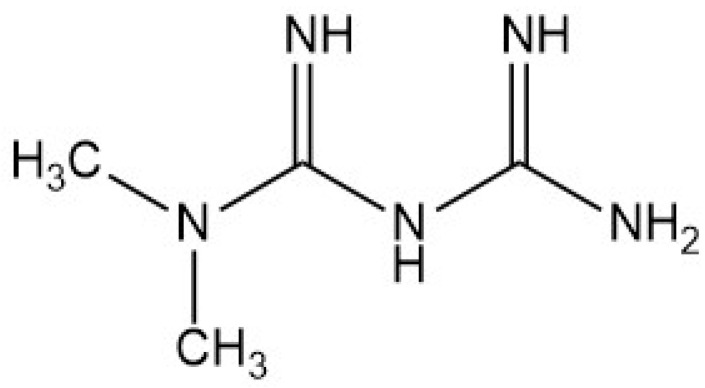
Structure of metformin (molecular weight: 129.167 g/mol; molecular formula: C_4_H_11_N_5_).

Metformin (MET), N,N-dimethylimidodicarbonimidic diamide, is one of the most common prescribed antidiabetic agents used for the treatment of type 2 diabetes mellitus (T2DM), or non-insulin-dependent diabetes. MET is an amino-group-rich compound with biguanide structures, containing two coupled molecules of guanidine with additional substitutions (Figure 2), which determine the blood-normalizing action of this drug. Physiologically, metformin directly or indirectly decreases glucose production in the liver, enhancing insulin sensitivity, and acts on the gut to increase glucose utilization [74]. MET can be used alone or in combination with other antidiabetic agents, such as sulfonylureas, alpha-glucosidase inhibitors or insulin [75]. It is an oral drug with a daily dosage ranging from 500 to 25,000 mg [76].

The worldwide consumption of metformin has resulted in the development of many electrochemical methods for its sensitive and selective determination in pharmaceuticals, biological fluids and environmental samples (Table 7). Based on a catalytic hydrogen evolution reaction with a hanging mercury drop electrode (HMDE), the quantitative determination of MET was possible in the linear range of 0.1 to 2 µM, and the LOD was equal to 0.018 µM [76]. As a result of the toxicity of mercury, environmentally friendly electrodes have been used more frequently. According to data from the literature, the most popular MET voltametric sensors are composed of a carbon paste electrode (CPE). Significant improvements in the selectivity and selectivity of CPEs have been achieved through the introduction of various kinds of nanomaterials, such as Fe-Cu/TiO_2_ [77], copper(II)-loaded activated charcoal [78], γ-Fe_2_O_3_@ hydrohyapatite/Cu(II) magnetic nanocomposites [79], sized mesoporous silica materials functionalized by copper ion [80], pyrogallol [81], molecular wires containing copper(II) and multiwalled carbon nanotubes [82], copper–graphene nanocomposites [83] and nickel oxide nanotube/carbon microparticle/Nafion composites [75]. Most of these constructions are based on the utilization of materials containing copper, which reacts with metformin, forming an electro-active complex determined voltammetrically. The catalytic action of cooper(II) ions on the electrochemical oxidation of metformin leads to fabrication, i.a. Fe-Cu/TiO_2_/CPE [77] and Cu-AC-CPE [78] are characterized by some of the lowest LOD values reported in the literature, equal to 3 nM and 9 nM, respectively. The possibility of the low-level determination of MET by these sensors has found applications in biological fluids, such as urine.

### 2.4. Dipeptidyl Peptidase-4 Inhibitor

The action of drugs from this group is to quickly and completely inhibit the activity of dipeptidyl peptidase-4 inhibitor (DPP-4), which contributes to the inactivation of endogenous glucagon-like peptide 1 (GLP-1). This causes an increase in the concentration of endogenous incretins, GLP-1 and GIP (gastric inhibitory peptide), on an empty stomach and after a meal. The effect on the pancreas is similar to the action of GLP-1 mimetics, but drugs from this group usually do not slow down emptying the stomach or clinically significant weight loss. They stimulate the secretion of insulin when needed (after a meal), have a protective effect on pancreatic β cells and have a positive effect on cholesterol levels. They are recommended for overweight and obese people and are intended for the treatment of type 2 diabetes. These drugs are administered orally, once a day at a fixed time, regardless of the meal. Gliptins can be used alone or in combination with metformin, sulfonylureas or thiazolidinedione derivatives [89,90,91,92,93,94,95,96].

#### 2.4.1. Sitagliptin

**Figure 3:**
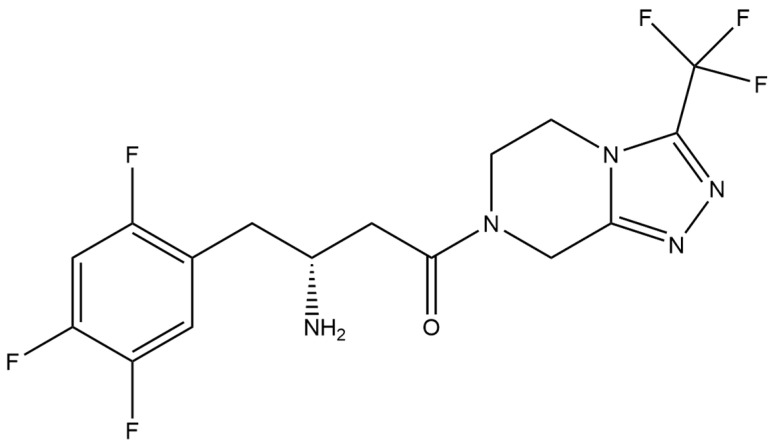
Structure of sitagliptin (molecular weight: 523.32 g mol^−1^; molecular formula: C_16_H_15_F_6_N_5_O).

Sitagliptin (STG) (Figure 3) inhibits the breakdown of incretin hormones in the body. These hormones stimulate the pancreas to produce insulin. By prolonging the action of incretin hormones in the blood, sitagliptin stimulates the pancreas to produce more insulin when glucose levels are high. It also reduces the amount of glucose produced by the liver by increasing insulin levels and decreasing the level of a hormone called glucagon. Together, these processes reduce blood glucose levels and help to control type 2 diabetes [97,98,99,100].

There are not many electrochemical assays of sitagliptin reported in the current literature (Table 8). Both of them use the differential pulse voltammetry technique as the determination method. The lowest obtained detection limit was equal to 0.06 pM on the screen-printed platinum electrode modified with molecularly imprinted polymer nanoparticles immobilized on its surface [101]. The proposed method has been successfully applied to the determination of STG in plasma samples. Another reported assay used a hanging mercury drop electrode as the working electrode, which allowed the authors to obtain LOD parameters as low as 2.6 nM, and the method has been applied to STG determination in pharmaceutical products [102].

#### 2.4.2. Linagliptin

**Figure 4:**
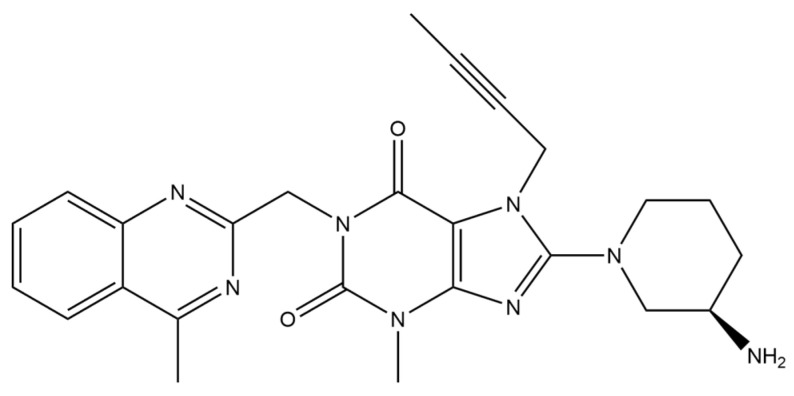
Structure of linagliptin (molecular weight: 472.54 g mol^−1^; molecular formula: C_25_H_28_N_8_O_2_).

Linagliptin (Figure 4) is an organic chemical compound from the group of dipeptidyl peptidase-4 inhibitors. It is used in type 2 diabetes to improve glycemic control. Linagliptin, in a glucose-dependent manner, increases insulin secretion and reduces glucagon secretion, thus allowing for an overall improvement in glucose homeostasis. Linagliptin can be used both in monotherapy and also in combination with metformin and sulfonylurea derivatives. The side effects of linagliptin intake may include nose and throat inflammation and, in some cases, angioedema, pancreatitis and joint pain. The administration of linagliptin is not recommended for women who are pregnant or breastfeeding.

Among the electrochemical methods for linagliptin determination, the most popular are voltametric techniques, such as differential pulse voltammetry and square-wave voltammetry (Table 9). In most cases, modified solid electrodes were chosen as the sensing elements. As a modifier, carbon nanotubes and metal oxides were commonly used. The lowest LOD was obtained for the carbon paste electrode modified with molecularly imprinted poly-itaconic and multiwalled carbon nanotubes, equal to 1 × 10^−13^ M [103]. The proposed method was successfully applied for highly sensitive linagliptin determination in complex matrices, such as pharmaceutical formulations in the form of tablets, urine and serum samples.

#### 2.4.3. Vildagliptin

**Figure 5:**
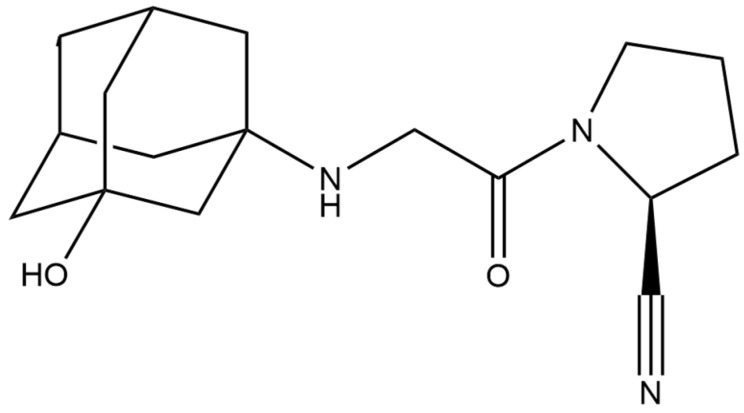
Structure of vildagliptin (molecular weight: 303.406 g mol^−1^; molecular formula: C_17_H_25_N_3_O_2_).

Vildagliptin (Figure 5) is an antidiabetic drug that belongs to the group of incretin drugs. Incretins are hormones produced in intestinal cells that, in response to the presence of food substances in the digestive tract, increase insulin secretion via Langerhans islets beta cells in the pancreas; these include glucagon-like peptide 1 (GLP-1) and glucose-dependent insulinotropic peptide (GIP, also called gastric inhibitory peptide). Incretin drugs mimic the action of incretins or increase their concentration by inhibiting the enzymes that break them down. Vildagliptin belongs to the second group mentioned. These are dipeptidyl peptidase IV (DPP-4) inhibitors, the so-called gliptin. They increase the concentration of incretins by inhibiting the enzyme that breaks down these hormones. There is an increase in the concentration of glucagon-like peptide 1 and glucose-dependent insulinotropic peptide, which improves the sensitivity of beta cells of the islets of Langerhans of the pancreas to glucose, increases insulin secretion and improves the activity of alpha cells responsible for the production of glucagon, without disturbing the glucagon response to hypoglycemia. Drugs from this group also do not cause weight gain. The indication for the use of vildagliptin is monotherapy for type 2 diabetes or combination therapy with another oral antidiabetic drug.

Vildagliptin was measured electrochemically, mainly using the square-wave voltammetry technique (Table 10). A wide variety of working electrodes was used, including boron-doped diamond electrodes, pencil graphite electrodes and platinum electrodes. Additionally, a modification of the carbon paste electrode was used in the form of mixing the carbon paste with calcium and montmorillonite clay. As a result, the lowest LOD obtained was equal to 77.52 nM [113]. Vildagliptin was successfully determined in matrices such as pharmaceutical formulations in the form of tablets, human serum and urine, and also in the cell lines.

### 2.5. Thiazolidinedione Derivatives

Thiazolidinedione derivatives (glitazones) are selective agonists of peroxisome proliferator-activated nuclear receptors (PPAR-γ), which are found mainly in adipose tissue, muscle and liver. The stimulation of these receptors leads to the transcription of genes responsible for the production, transport and metabolism of glucose and fatty acids. Glitazones reduce both fasting and postprandial glycemia without the risk of hypoglycemia. The favorable metabolic profile of these drugs results from increased tissue sensitivity to insulin, reduced insulin resistance in adipose tissue and a decrease in the concentration of free fatty acids and glucose in the blood [118,119,120,121,122].

#### 2.5.1. Pioglitazone

**Figure 6:**
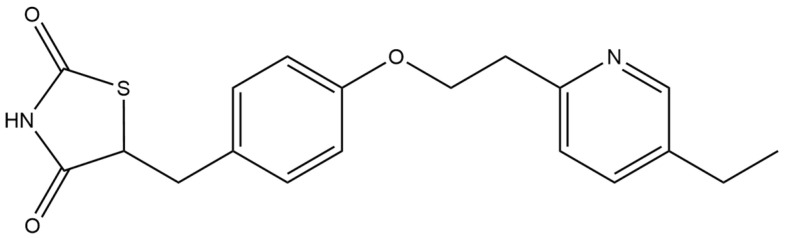
Structure of pioglitazone (molecular weight: 356.44 g mol^−1^; molecular formula: C_19_H_20_N_2_O_3_S).

Pioglitazone (PIO) (Figure 6) improves the sensitivity of peripheral tissues to insulin, controls glycemia, dyslipidemia and hypertension, and also reduces albuminuria in patients with type 2 diabetes. Reducing glycemia, both fasting and postprandial, occurs mainly due to hepatic and peripheral (muscle) reduction in insulin resistance. Moreover, pioglitazone has proven to be effective not only in the treatment of type 2 diabetes, but also in preventing its development. Pioglitazone activates peroxisome proliferator-activated receptors (PPAR-γ), and causes a decrease in the insulin resistance of muscle and adipose tissue and a decrease in gluconeogenesis in the liver. The effect of pioglitazone is to reduce glycemia, insulinemia and triglycerides, and increase the HDL cholesterol fraction in the blood. Pioglitazone does not increase insulin secretion and acts only in its presence, and does not cause hypoglycemia [123,124,125,126,127,128,129].

A variety of electrochemical techniques among the different types of working electrodes were implemented for highly sensitive pioglitazone determination (Table 11). Aside from the classic construction of the hanging mercury drop electrode, mainly solid electrodes and carbon paste electrodes were used for this matter. The lowest reported PIO detection limit was obtained using the HMDE electrode, and it was equal to 8.08 nM. Other reported assays include the usage of glassy carbon electrodes, on which the lowest LOD was of about 0.07 µM, or screen-printed graphite electrodes, with the LOD parameter equal to 29 nM. In order to check the possibilities of the proposed methods for routine quality control analysis, PIO measurements were performed on pharmaceuticals and serum samples.

#### 2.5.2. Rosiglitazone

**Figure 7:**
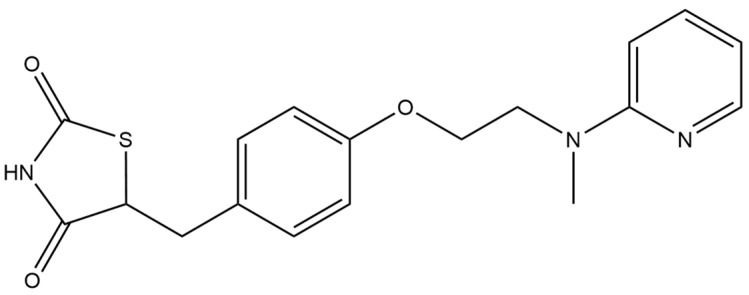
Structure of rosiglitazone (molecular weight: 357.43 g mol^−1^; molecular formula: C_19_H_20_N_2_O_3_S).

Rosiglitazone (Figure 7) is an organic chemical compound, a drug used in the treatment of diabetes, belonging to the thiazolidinedione group of oral hypoglycemic drugs. It is a selective agonist of the nuclear peroxisome proliferator-activated receptor γ (PPAR-γ), located at the border of the cell nucleus and cytoplasm. Activation of the PPAR-γ receptor leads to the transcription of genes involved in the synthesis, transport and utilisation processes of glucose and the regulation of the metabolism of fatty acids. By acting on PPAR-γ, rosiglitazone reduces insulin resistance in adipocytes, skeletal muscle myocytes and hepatocytes. The drug reduces insulinemia, the need for endogenous insulin and the concentration of free fatty acids and glucose in the blood.

Among the reported methods of rosiglitazone determination, a few voltametric assays were presented (Table 12). As the working electrode, classic mercury electrodes were used, including dropping mercury electrodes or the hanging mercury drop electrodes. In addition, carbon electrodes, such as glassy carbon electrodes and carbon paste electrodes, were used in this respect. Rosiglitazone was successfully measured in matrices such as pharmaceutical formulations, human urine and plasma. Under optimised conditions, the LOD of rosiglitazone was specified as 3.2 × 10^−11^ M.

### 2.6. Repaglinide

**Figure 8:**
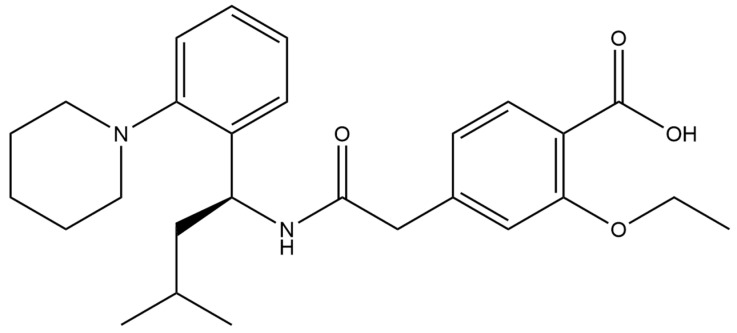
Structure of repaglinide (molecular weight: 452.595 g mol^−1^; molecular formula: C_27_H_36_N_2_O_4_).

Repaglinide (Figure 8) is an organic chemical compound, an antidiabetic and hypoglycemic drug. It is a short-acting hypoglycemic drug that belongs to carbamoylbenzoic acid derivatives from the group of drugs known as meglitinides, which were invented in 1983. Its proven mechanism of action is to stimulate insulin release from pancreatic β cells by inhibiting ATP-dependent potassium channels. The main side effect is the possibility of hypoglycemia. In type 2 diabetes, this drug quickly corrects the disorder of meal-stimulated insulin secretion, without increasing the secretion of the hormone between meals and at night. This allows a patient treated with repaglinide to eat meals in a rhythm that is customised to individual needs while reducing the risk of hypoglycemia.

Differential pulse and square-wave voltammetry was successfully applied to the highly sensitive determination of repaglinide, mostly in pharmaceutical samples in the form of tablets, but studies on serum and urine were also performed (Table 13). However, bare glassy carbon electrodes and carbon paste electrodes were successfully used for repaglinide determination [135,136]; the usage of modification layers led to obtaining better results considering the linearity range and limits of detection. The lowest LOD was obtained using glassy carbon electrodes modified with a composite of three-dimensional porous reduced graphene oxide nanostructures and SnO_2_ nanoparticles [137].

## 3. Conclusions

Pharmacotherapy for diabetes with injective and oral hypoglycemic drugs has never been as broad and effective as it is today. It allows for the individualisation of treatment in most patients, the gain of control over the disease and the prevention of its complications. This review summarises the current state of knowledge in the field of electrochemical techniques used for determination of antidiabetic active substances in different matrices. Due to their simplicity, portability and easiness, electrochemical sensors have found a unique place in this matter, and the number of new proposed assays is still growing. It has also been proven that the proposed methods are suitable for the quality control analysis of pharmaceutical products and, in some cases, for monitoring drug concentrations in human bodily fluids.

## Figures and Tables

**Table 1:** Electrochemical methods of insulin determination.

Electrode	Technique	Medium	Linear Range	LOD	Samples	Ref.
CNT-NiCoO_2/_Nafion	Amperometry	0.1 M PBS pH 7.5	0.1–31.5 µg mL^−1^	0.22 µg mL^−1^	-	[21]
CHN|CCE	FIA	0.3 M PBS pH 10	0.5–15 nM	0.11 nM	Human serum	[27]
GCE-IrOx	Amperometry	0.10 mM Na_3_IrCl_6_ + 0.20 M HCl pH 7.4	0.05–0.50 µM	20 nM	-	[22]
CCE[Ru(bpy) (tpy)CI]PF_6_	Amperometry	0.1 M PBS pH 7.0	0.5–850 nM	0.4 nM	-	[28]
CPE/RuOx	FIA	0.1 M NaCl + 0.05 M phosphate buffer pH 7.4	100–1000 nM	50 nM	-	[29]
GCE/RuOx-CNT	FIA	0.05 M PBS pH 7.4	10–800 nM	1 nM	-	[30]
SPE/NiONPs/Nafion-MWCNTs	Amperometry	0.1 M NaOH pH 13	20–260 nM	6.1 nM	-	[31]
GCE/SiC	Amperometry	PBS pH 7.4	0.1–0.6 nM	0.0033 nM	-	[32]
Si-CPE	Amperometry	PBS pH 2.0	90–1400 pM	36 pM	-	[33]
GCE/RuRDMs	FIA	0.2 M PBS pH 7.0	6–400 nM	2 nM	-	[34]
GCE/CHIT-CNT	Amperometry	PBS pH 7.4	0.1–3.0 µM	30 nM	-	[35]
ITO/NiNPs	CV	0.1 M NaOH	1–125 nM	10 nM	Bovine insulin injections	[24]
NiNPs/CNTs/CFME	CV	0.1 M NaOH	2–20 µM	270 nM	-	[36]
SiO_2_ NPs-Nafion/GCE	DPV	0.1 M PBS pH 7.35	10–50 nM	3.1 nM	Injections, skin sweat	[37]
CNT/GC	FIA	0.05 M PBS pH 7.4	100–1000 nM	14 nM	-	[38]
GC/rGO	CV	0.1 M PBS pH 7.4	4–640 nM	350 pM	Human serum	[39]
Guanine/NiOx-GC	Amperometry	PBS pH 7.4	100 pM–4 µM	22 pM	-	[23]
Ni(OH)_2_NPs/Nafion-MWCNTs/GC	Amperometry	0.1 M NaOH	0–40 µM	85 nM	Pharmaceuticals, human plasma	[40]
PGE/NiNPs/MB	SWV	B-R pH 7.0	25–450 nM	33.17 nM	Human serum	[41]
MWNTs/DMF/TFT	CV	0.05 M PBS pH 7.4	250 nM–1.6 µM	250 nM	-	[42]
GCE/CoOx	FIA	PBS pH 9.0	100 pM–15 nM	25 pM	-	[43]
SPCE/MWCNT/NiO1.5	Amperometry	PBS + 0.1 M NaOH	600 nM–10 µM	19.6 nM	Bovine blood serum	[44]
MIP-SPCE	SWV	0.01 M PBS pH 7.2	20–70 pM	1.9 pM	Pharmaceutical samples	[25]
NanoMIP/SPPE	DPV	5 mM PBS pH 7.2	50–2000 pM	26 fM	Human plasma	[45]
Aptamer/cDNA- MSTF	DPV	5 mM Fe(CN)_6_^−3/−4^	10–350 nM	3 nM	Biological samples	[46]
Au/PPy/AuNPs/L-cys/ZIF-8	SWV	pH 7.0	1–60 nM	1 nM	Pharmaceutical samples, human serum	[47]
CNPE/DNA	SWV	0.1 M NH_4_H_2_PO_4_ pH 4.5	0.01–0.1 ng L^−1^	0.004 ng L^−1^	Pharmaceutical samples	[48]
AgNF/rGO/MDEA	EIS	PBS	1–1000 ng mL^−1^	70 pg mL^−1^	-	[26]
GC/Ni(OH)_2_-GN	CV	0.1 M NaOH pH 11	800–6400 nM	200 nM	Artificial physiological matrix, spiked human serum	[49]
PGE/GNPs/ss-DNA	EIS	PBS pH 7.4 + 10 mM MgCl_2_	1–1000 nM	50 nM	Plasma and urine	[50]
CPE/RBC	Amperometry	PBS pH 7.4	0.006–0.09 µM	0.006 µM	Human serum	[51]

CNT—carbon nanotube; CNPE/DNA—carbon nanotube paste electrode graphite powder with DNA; CHN|CCE—carbon ceramic electrode modified with cobalt hydroxide nanoparticles; IrOx—iridium oxide; CCE—carbon composite ceramic electrode; NiONPs/Nafion-MWCNTs/SPE—screen-printed electrode modified with nickel oxide nanoparticles and Nafion-multiwalled carbon nanotubes; GCE/SiC—glassy carbon electrode modified with silicon carbide nanoparticles; Si-CPE—silica gel-modified carbon paste electrode; GCE/RuRDMs—glassy carbon electrode modified with ruthenium metallodendrimer multilayers; GCE/CHIT-CNT—glassy carbon electrode coated with chitosan films and multiwalled carbon nanotubes; NiNPs/ITO—nickel nanoparticles modified with indium tin oxide electrode; NiNPs/CNTs/CFME—nickel nanoparticle carbon nanotube-modified carbon fiber microelectrode; SiO_2_ NPs-Nafion/GCE—silica nanoparticles/Nafion-modified glassy carbon electrode; Ni(OH)_2_NPs/Nafion-MWCNTs/GC—glassy carbon electrodes modified with Nafion-multiwalled carbon nanotubes decorated with nickel hydroxide nanoparticles; PGE/NiNPs/MB—Ni nanoparticles and methylbenzoate-modified pencil graphite electrode; MWNTs/DMF/TFT—carbon electrode modified using standard thick-film technology with planar multiwalled carbon nanotubes; SPCE/MWCNT/NiO1.5—screen-printed carbon electrode with electrodeposited NiO nanoparticles; NanoMIP/SPPE—screen-printed platinum electrodes modified with molecularly imprinted polymer nanoparticles; Aptamer/cDNA-Gated amine functionalized MSTF—functionalized mesoporous silica thin film coated on a glassy carbon electrode; Au/PPy + Au NPs/l-cysteine/ZIF-8 crystalline—Au bare electrode modified with zeolitic imidazolate framework-8; CNPE/DNA—DNA immobilized onto a carbon nanotube paste electrode; CNPE/DNA—label-free impedimetric biosensor based on the easy immobilization of an antibody bioreceptor on AgNF-rGO nanostructured ITO micro-disk electrode; AgNF/rGO/MDEA—Ag nanoflower-decorated, reduced graphene oxide-modified micro-disk electrode arrays; GC/Ni(OH)_2_-GN—electrochemically active nickel hydroxide–graphene nanocomposites; PGE/GNPs/ss-DNA—a poly-orthophenylene diamine substrate decorated with gold nanoparticles and single-stranded DNA aptamer immobilized on the pencil graphite electrode; CPE/RBC—carbon paste electrodes with red blood cells.

**Table 2:** Comparison of sulfonylurea agents.

Molecule	IUPAC Name	Molar Mass, g mol^−1^	Generation	Dose, mg	Structure
Tolbutamide	1-butyl-3-(4-methylphenyl)sulfonylurea	270.35	I	500–2000	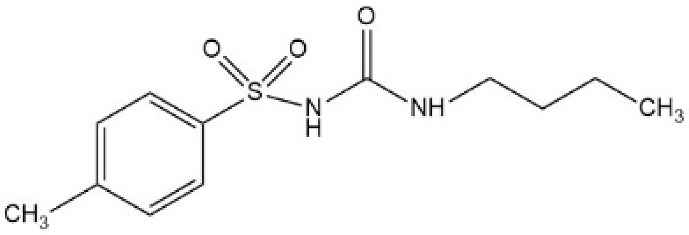
Gliclazide	1-(3,3a,4,5,6,6a-hexahydro-1H-cyclopenta[c]pyrrol-2-yl)-3-(4-methylphenyl)sulfonylurea	323.4	II	40–320	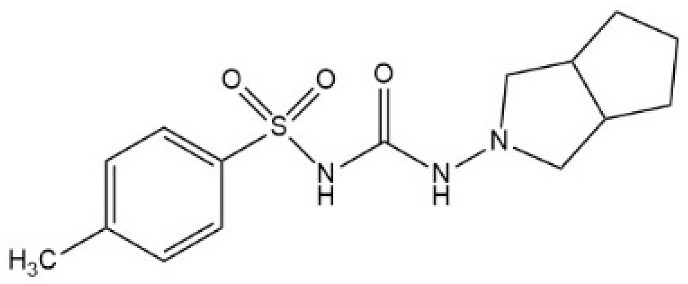
Glipizide	N-[2-[4-(cyclohexylcarbamoylsulfamoyl)phenyl]ethyl]-5-methylpyrazine-2-carboxamide	445.5	II	2.5–20	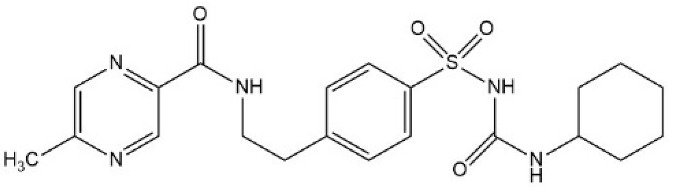
Glibenclamide	5-chloro-N-[2-[4-(cyclohexylcarbamoylsulfamoyl)phenyl]ethyl]-2-methoxybenzamide	494	II	2.2–15	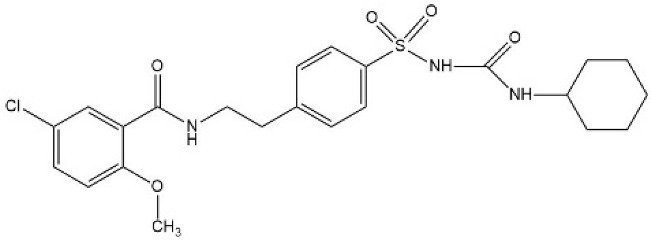
Glimepiride	4-ethyl-3-methyl-N-[2-[4-[(4-methylcyclohexyl)carbamoylsulfamoyl]phenyl]ethyl]-5-oxo-2H-pyrrole-1-carboxamide	490.6	II	1–6	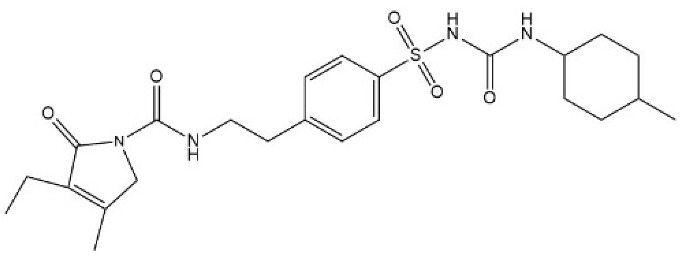

**Table 3:** Electrochemical methods of gliclazide determination.

Electrode	Technique	Medium	Linear Range, mol L^−1^	LOD, mol L^−1^	Samples	Ref.
CPE	DPV	0.04 M B-R buffer pH 4.0	5 × 10^−7^–1.25 × 10^−6^	1 × 10^−7^	Tablets	[54]
E-MIP-GCE	DPV	0.2 M PBS pH 5.0	5 × 10^−11^–4 × 10^−10^	1.2 × 10^−11^	Tablets, urine	[55]
Fe_3_O_4_@SiO_2_/MWCNT/GCE	DPV	0.1 M PBS pH 7.0	5 × 10^−6^–8.0 × 10^−4^	2.1 × 10^−6^	Tablets, urine	[56]
ZISILCPE	DPV	0.1 M PBS pH 7.0	7.5 × 10^−7^–5.0 × 10^−4^	1.2 × 10^−7^	Pharmaceuticals, urine	[57]
Fe_3_O_4_NP/SPE	DPV	PBS pH 7.0	5.0 × 10^−7^–6.0 × 10^−4^	1 × 10^−7^	Pharmaceuticals, urine	[60]
MoWS2/SPE	DPV	0.1 M PBS pH 7.0	6.0 × 10^−8^–5.0 × 10^−4^	1.8 × 10^−8^	Tablets, urine	[58]
MCSNP/SPCE	SWV	0.1 M PBS pH 7.0	5.0 × 10^−7^–3.0 × 10^−4^	1 × 10^−7^	Tablets, urine	[59]

CPE—Carbon paste electrode; HDME—hanging mercury drop electrode; GCE—glassy carbon electrode; E-MIP-GCE—electropolymerized molecularly imprinted polymer film onto GCE; Fe_3_O_4_@SiO_2_/MWCNT/GCE—magnetic core–shell Fe_3_O_4_@SiO_2_ and multiwalled carbon nanotube-modified glassy carbon electrode; ZISILCPE—ZnIn_2_S_4_ nanoparticle (ZIS)- and ionic liquid (IL)-modified carbon paste electrode; Fe_3_O_4_NP/SPE—Fe_3_O_4_ nanoparticle-modified screen-printed electrode; MoWS2—modified screen-printed electrode; MCSNP/SPCE—magnetic core–shell manganese ferrite nanoparticle-modified screen-printed electrode;; CV—cyclic voltammetry.

**Table 4:** Electrochemical methods of glipizide determination.

Electrode	Technique	Medium	Linear Range, mol L^−1^	LOD, mol L^−1^	Samples	Ref.
CPE	DPV	B-R buffer pH 5.0	7.5 × 10^−7^–5 × 10^−5^	2.5 × 10^−5^	pharmaceutical formulation	[62]
HMDE	SWAdCSV	B-R buffer pH 6.0	5 × 10^−10^–1 × 10^−8^	1.5 × 10^−10^	pharmaceutical formulation	[61]
in situ plated lead film electrode	SWAdS	0.1 M acetate buffer pH 4.7	5 × 10^−10^–1 × 10^−8^	2.5 × 10^−10^	pharmaceutical formulation, human urine	[64]

**Table 5:** Electrochemical methods of glibenclamide determination.

Electrode	Technique	Medium	Linear Range, mol L^−1^	LOD, mol L^−1^	Samples	Ref.
SMCPE	DPV	0.04 M B-R buffer, pH 5.0	1.0 × 10^−9^–5.0 × 10^−8^	4 × 10^−10^	Tablets, human serum	[66]
ZISILCPE	DPV	0.1 M PBS, pH 7.0	1.0 × 10^−6^–8.0 × 10^−4^	8 × 10^−7^	Tablets, urine	[57]
HMDE	SWAdCSV	0.04 M B-R buffer, pH 9.7	2 × 10^−8^–1 × 10^−6^	6 × 10^−9^	Diabetic drugs, human serum	[65]

**Table 6:** Electrochemical methods of glimepiride determination.

Electrode	Technique	Medium	Linear Range	LOD	Samples	Ref.
GCE	DPV	phosphate buffer pH 6.0	1 × 10^−5^–3.2 × 10^−5^ mol L^−1^	2 × 10^−6^ mol L^−1^	Tablets	[67]
CPE			2 × 10^−6^–1.5 × 10^−5^ mol L^−1^	7.5 × 10^−7^ mol L^−1^		
CPE	DPV	B-R buffer pH 6.0	1.5 × 10^−6^–3 × 10^−6^ mol L^−1^	2 × 10^−7^ mol L^−1^	Tablets	[68]
GCE			1.5 × 10^−6^–4 × 10^−6^ mol L^−1^	6 × 10^−7^ mol L^−1^		
HMDE	SVW	0.04 M B-R buffer pH 9.0	0.25–7.81 µg mL^−1^	0.09 µg mL^−1^	Tablets	[70]
HMDE	SWAdCSV	0.04 M B-R buffer pH 9.0	1.0–10.0 µg mL^−1^	2.4 µg mL^−1^	Environmental water	[72]
HMDE	SWV	phosphate buffer pH 7.0	-	3.48 × 10^−8^ mol L^−1^	n/i	[73]
GCE	CV	0.1 M acetate buffer pH 6.8	-	1.7 × 10^−5^ mol L^−1^	n/i	[69]
GRE			-	3.51 × 10^−5^ mol L^−1^	n/i	

**Table 7:** Electrochemical methods of metformin determination.

Electrode	Technique	Medium	Linear Range	LOD	Samples	Ref.
GNF-PMB/SnO_2_/F	CV	SPB, pH 7.0, 0.05 M	0.01–1 mM	0.1 nM	Urine, plasma	[84]
Fe-Cu/TiO_2_/CPE	SWAdSV	Phosphate buffer, pH 12, 0.1 M	0.015–75 μM	3 nM	Pharmaceutics, urine	[77]
MIP-AgNPs-PGE	DPV	Britton–Robinson buffer, pH 4.0	0.1–10 μM	6.8 nM	Pharmaceutics, plasma	[85]
Cu-AC-CPE	DPV	Phosphate buffer, pH 12, 0.1 M	0.05–60 μM	9 nM	Pharmaceutics, urine	[78]
γ-Fe_2_O_3_@HAp/Cu(II)-CPE	AdDPV	Phosphate buffer, pH 12, 0.1 M	0.1–80 μM	0.014 μM	Pharmaceutics, urine	[79]
CGMDE	SWV	Acetate buffer pH 4.7, 0.01M	0.1–2 μM	0.018 μM	Urine	[86]
	DPV		0.1–2 μM	0.032 μM	-	
	LSV		0.2–2 μM	0.077 μM	-	
SBA-15-Cu(II)/CPE	DPV	Phosphate buffer, pH 12, 0.1 M	0.1–65 μM	0.03 μM	Pharmaceutics, urine, plasma	[80]
PYCPE	DPV	Britton–Robinson, pH 2.0	0.8–6 μM	0.0663 μM	Pharmaceutics, urine	[81]
MWCNT/PE	LSV	Ammonia buffer, pH 8.9, 0.1M	0.2–10 μM	0.067 μM	Pharmaceutics	[87]
CB-DHP/GCE	DPV	Acetate buffer, pH 4.5, 0.1M	2–10 μM	0.63 μM	Sewage	[88]
CuMW/CNT/PE	SSV	Britton–Robinson, pH 7.2	0.9–50 μM	0.65 μM	Pharmaceutics	[82]
Cu-G/CPE	DPV	PBS, pH 12, 0.1M	10.4–1125 μM	3.4 μM	Pharmaceutics, plasma	[83]
CB/RuO_2_/Nafion- GCE	DPV	Acetate buffer, pH 4.6, 0.05 M	10–70 μM	0.7 μM	Pharmaceutics, sewage, river water	[76]
n-NC/CPE	chronoamperometry	NaOH, 100 mM	4–63 μM	0.45 μM	Plasma, urine, breast milk	[75]

GNF-PMB/SnO_2_/F—graphene nanoflakes–polymethylene blue nanocomposite developed onto fluorine-doped tin oxide glass electrode; Fe-Cu/TiO_2_/CPE—carbon paste electrode modified with Fe-Cu/TiO_2_ nanocomposite; MIP-AgNPs-PGE—pencil graphite electrode modified with molecularly imprinted polymer and silver nanoparticles; Cu-AC-CPE—carbon paste electrode modified with copper(II)-loaded activated charcoal; γ-Fe_2_O_3_@HAp/Cu(II)-CPE—carbon paste electrode modified with γ-Fe_2_O_3_@ hydrohyapatite/Cu(II) nanocomposite; CGMDE—controlled-growth mercury drop electrode; SBA-15-Cu(II)/CPE—carbon paste electrode modified with nano-sized mesoporous silica material functionalized by copper ion; PYCPE—carbon paste electrode modified with pyrogallol; MWCNT/PE—multiwalled carbon nanotube paste electrode; CB-DHP/GCE—glassy carbon electrode modified with a carbon black dihexadecylphosphate film; CuMW/CNT/PE—paste electrode modified with molecular wires containing copper(II) and multiwalled carbon nanotubes; Cu-G/CPE—carbon paste electrode modified with copper–graphene nanocomposite; CB/RuO_2_/Nafion-GCE—glassy carbon electrode modified with carbon black, RuO_2_∙xH_2_O and Nafion; n-NC/CPE—carbon paste electrode modified with nickel oxide nanotubes/carbon microparticles/Nafion nanocomposite; SSV—single sweep voltammetry.

**Table 8:** Electrochemical methods of sitagliptin determination.

Electrode	Technique	Medium	Linear Range	LOD	Samples	Ref.
nanoMIP-SPPE	DPV	PBS buffer, 5 mM, pH 7.2	100–2000 pM	0.06 pM	plasma	[101]
Hg(Ag)FE	DPV	Ammonium buffer, 0.025 M, pH 8.2	0.02–0.14 μM	2.6 nM	pharmaceutics	[102]

nanoMIP-SPPE—molecularly imprinted polymer nanoparticles immobilized on screen-printed platinum electrode; Hg(Ag)FE—renewable amalgam film electrode.

**Table 9:** Electrochemical methods of linagliptin determination.

Electrode	Technique	Medium	Linear Range	LOD	Samples	Ref.
GC/GrOx	SWV	40 mM phosphate buffer, pH 6.5	9.45–103.96 ng mL^−1^	4.0 ng mL^−1^	Tablet formulations, spiked human urine, plasma, rats’ feces	[104]
CPE/gCN-βCD	DPV	0.1 M phosphate buffer, pH 7.0	0.01–50 µM	3 nM	Blood serum	[105]
PGE/Cu microparticles	SWV	0.04 M B-R buffer pH 4.5	47.25–283.53 ng mL^−1^	47.25 ng mL^−1^	Spiked urine and plasma sample, tablets	[106]
PGE	SWV	Teorell–Stenhagen buffer, pH 5.5, + 0.1 M NaClO_4_	0.24–5.20 μg mL^–1^	0.10 μg mL^–1^	Tablets, spiked human urine and plasma	[107]
L-cys@MoS_2_/GCE	DPV	0.25 M B-R buffer, pH 7.0	1.0–153.4 µM	0.19 µM	Plasma	[108]
CPE/Fe_2_O_3_NPs	SWV	B-R buffer, pH 7.4	0.03–86 μg mL ^–1^	8.0 ng mL^–1^	Tablets, spiked urine	[109]
MWCNT/MIP/CPE	DPV	0.1 M B-R buffer, pH 8.0	1 × 10^−12^–1 × 10^−7^ M	1 × 10^−13^ M	Tablets, urine, serum	[103]
PGE	DPV	0.5 M acetate buffer, pH 4.8	100–600 μg mL^−1^	21.5 μg mL^−1^	-	[110]
GCE/E-rGO/Poly (β-CD)/magnetic ZIF-67	DPV	B-R buffer, pH 7.0	0.03–200 μM	0.01 μM	Human plasma and urine	[111]
	amperometry		0.02–300 μM	0.006 μM	-	
Co_3_O_4_NPs/MWCNTs/CPE	SWV	B-R buffer, pH 8.0	3.98 × 10^−5^–1.53 × 10^−3^ M	1.13 × 10^−5^ M	Tablets, urine	[112]

Co_3_O_4_NPs/MWCNTs/CPE—cobalt oxide nanoparticles and multiwalled carbon nanotube-modified carbon paste electrode; GCE/E-rGO/Poly (β-CD)/magnetic ZIF-67—glassy carbon electrode modified with graphene, β-cyclodextrin and magnetic ZIF-67; L-cys@MoS2/GCE—L-cysteine-decorated MoS2-modified glassy carbon electrode; CPE/gCN-βCD—graphitic carbon nitride/β-cyclodextrin nanocomposite; GC/GrOx—graphene oxide-modified glassy carbon electrode.

**Table 10:** Electrochemical methods of vildagliptin determination.

Electrode	Technique	Medium	Linear Range	LOD	Samples	Ref.
BDDE	SWV	B–R buffer + 0.5 mM SDS, pH 11.0	2.94–55.86 µM	77.52 nM	Tablets, urine	[113]
PGE	SWV	PBS, pH 9.0	2.94–49.98 µM	82 nM	Tablets, urine	[114]
Pt	LSV	Phosphate buffer, pH 6.8	2–10 mM	0.241 mM	Tablets	[115]
Ca-MMT/CPE	SWV	B–R buffer, pH 7.0	4.0–130 nM	1.19 nmol L^−1^	Tablets, cell lines	[116]
Ca-MMT/CPE	SWV	B–R buffer, pH 7.0	1.0–110 nM	0.285 nmol L^−1^	Tablets, spiked human serum	[117]

Ca-MMT/CPE—carbon paste electrode modified with Ca-montmorillonite clay.

**Table 11:** Electrochemical methods of pioglitazone determination.

Electrode	Technique	Medium	Linear Range	LOD	Samples	Ref.
HMDE	SWAdSV	Britton–Robinson buffer, pH 5.0	0.01–100 µM	8.08 nM	Pharmaceutics, urine, serum	[130]
GCE	DPV	Britton–Robinson buffer, pH 6.0	1.5–3.0 µM	0.07 µM	Pharmaceutics	[68]
CPE				0.3 µM		
GCE	DPV	Phosphate buffer, pH 3.16, 0.2 M	6–100 µM	1.66 µM	Pharmaceutics	[131]
	SWV			1.12 µM		
Nr-GO/GCE	DPV	PBS, pH 7.0, 0.1 M	4–40 µM	67 nM	Synthetic solution	[132]
SPGE			4–60 µM	29 nM		

HMDE—hanging mercury drop electrode; GCE—glassy carbon electrode; CPE—carbon paste electrode; Nr-GO/GCE—glassy carbon electrode modified with nitrogen-doped reduced graphene oxide; SPGE—screen-printed graphite electrode.

**Table 12:** Electrochemical methods of rosiglitazone determination.

Electrode	Technique	Medium	Linear Range	LOD	Samples	Ref.
DME	DPP	0.08 M B–R buffer, pH 4.0	0.1–16 mg mL^−1^	0.07 mg mL^−1^	Tablets, spiked human plasma	[133]
DME	DC_t_		4–24 mg mL^−1^	0.15 mg mL^−1^	Tablets	
HMDE	SWV	B–R buffer, pH 5.0	5 × 10^−8^–8 × 10^−7^ M	3.2 × 10^−11^ M	Human urine and plasma	[134]
CPE	DPV	B–R buffer, pH 5.0	1.5–4 µM	5 × 10^−8^ M	Tablets	[68]
GCE			1.5 × 10^−6^–4 × 10^−6^ M	1 × 10^−7^ M		

DME—dropping mercury electrode, HMDE—hanging mercury drop electrode.

**Table 13:** Electrochemical methods of repaglinide determination.

Electrode	Technique	Medium	Linear Range	LOD	Samples	Ref.
CPE	DPV	B-R buffer, pH 6.0	8.0 × 10^−7^–3.2 × 10^−6^ M	1.348 × 10^−7^ M	Tablets	[135]
GCE	DPV	B-R buffer, pH 7.0	4.0 × 10^−7^–4.0 × 10^−6^ M	1.062 × 10^−7^ M	Tablets	
GCE	DPV	0.2 M H_2_SO_4_	1.81–90.58 µg mL^−1^	0.278 µg mL^−1^	Tablets	[136]
	SWV			0.230 µg mL^−1^	Tablets	
SnO_2_@p-rGO/GCE	DPV	Phosphate buffer, pH 3.0	4.99 × 10^−8^–1.83 × 10^−5^ M	9.02 × 10^−9^ M	Tablets	[137]
	SWV		1.99 × 10^−8^–1.45 × 10^−5^ M	0.85 × 10^−9^ M	Tablets	
MIP-PoDB/PoPD-GCE	DPV	5 mM [Fe(CN)_6_]^3−^/^4−^	0.005–1.0 µM	1.8 nM	Tablets, blood serum, urine	[138]

SnO_2_@p-rGO/GCE—composite of three-dimensional, porous, reduced-graphene-oxide nanostructure SnO_2_ nanoparticles on the glassy carbon electrode; MIP-PoDB/PoPD-GCE—glassy carbon electrode modified with electro polymerization of o-Dihydroxybenzene and o-phenylenediamine.

## Data Availability

No new data were created or analysed in this study. Data sharing is not applicable to this article.
